# The role of mesencephalic aqueduct obstruction in hydrocephalus development: a case report

**DOI:** 10.3325/cmj.2021.62.411

**Published:** 2021-08

**Authors:** Milan Radoš, Darko Orešković, Marijan Klarica

**Affiliations:** 1 Croatian Institute for Brain Research, Zagreb University School of Medicine, Zagreb, Croatia; 2 Department of Molecular Biology, Ruđer Bošković Institute, Zagreb, Croatia; 3 Department of Pharmacology, Zagreb University School of Medicine, Zagreb, Croatia

## Abstract

We report on three patients with mesencephalic aqueduct obstruction, which completely blocked the cerebrospinal fluid communication between the third and fourth cerebral ventricle, demonstrated by standard and high-resolution magnetic resonance sequences. Only one patient developed radiological and clinical presentation of hydrocephalus, without radiological signs of increased intraventricular pressure. The remaining two patients did not show clinical signs of hydrocephalus and had a normal radiological presentation of the ventricular system. These findings contradict the classical concept of cerebrospinal fluid physiology. This concept assumes a unidirectional circulation of cerebrospinal fluid through the mesencephalic aqueduct from the secretion site, predominantly in the choroid plexuses, to the resorption site, predominantly in the dural venous sinuses. Therefore, the obstruction of the mesencephalic aqueduct would inevitably lead to triventricular hypertensive hydrocephalus in all patients. The current observations, however, accord with the new concept of cerebrospinal fluid physiology, which postulates that cerebrospinal fluid does not circulate unidirectionally because it is both formed and resorbed along the entire capillary network within the central nervous system.

The generally accepted classical concept of cerebrospinal fluid (CSF) physiology postulates the mesencephalic aqueduct's patency as one of the preconditions for maintaining physiological volumes and pressures in the CSF system. According to the classical concept, CSF is secreted predominantly by the choroid plexuses within the ventricular system. It then flows unidirectionally into the subarachnoid space at the skull base, from where it circulates to the resorption site, located predominantly in the dural sinuses and to a lesser extent in the perineural lymphatic system (1-7). The CSF is thought to be actively secreted by the choroid plexuses at an approximate rate of 20 mL/h (8,9). Therefore, even a short-term obstruction of the mesencephalic aqueduct inevitably leads to the development of triventricular hypertensive hydrocephalus, which has a characteristic neuroradiological (dilation of the third cerebral ventricle and lateral ventricles with transependymal CSF edema) and clinical presentation (headache, nausea, visual disturbance, drowsiness, balance disorder).

In this study, we report on three patients of different ages with mesencephalic aqueduct obstruction. We analyzed their clinical and neuroradiological findings from the perspective of the classical concept of CSF physiology (1-7) and the Bulat-Orešković-Klarica hypothesis (10-14).

## PATIENT 1

Patient 1 was a male preterm infant born at a gestational age of 25 weeks and 2 days (birth weight 740 g, Apgar 5/7/8, head circumference 23.5 cm) (Table 1). Enteral feeding with breast milk was started from the third day and was well tolerated. At hospital discharge at the corrected age of 36 weeks and 6 days, he had a good weight gain (2590 g, 30 centiles) and normal head circumference (31 cm, 30 centiles). Ultrasound exam showed grade II periventricular hemorrhage, a consequence of preterm birth. Ophthalmological exam demonstrated retinopathy of prematurity stage II. Neuropediatric and neuropsychological exam at a corrected age of 24 months showed psychomotor development in the broader range of normal (average for gross motor tasks, lower average for fine motor skills, cognition. and speech).

**Table 1:** Patients' clinical status, diagnostic procedures, treatment and clinical outcomes

**Patient 1**	
Clinical status	premature birth, grade II periventricular hemorrhage, retinopathy of prematurity stage II
Gestational age at birth	25 weeks and 2 days
Apgar score	5/7/8
Birth weight	740 g
Treatment	non-invasive mechanical ventilation for 57 days, 14 days of high-flow nasal cannula, i.v. parenteral nutrition for the first 18 days, enteral intake of breast milk from the third day
First magnetic resonance (MR) exam	term-equivalent age
Second MR exam:	2 years
Clinical outcome	at a corrected age of 24 months, psychomotor development was in the broader range of normal (average for gross motor tasks, lower average for fine motor skills, cognition, and speech)
**Patient 2**	
Clinical status	premature birth, respiratory distress syndrome, sepsis development
Gestational age at birth	28 weeks and 6 days
Apgar score	6/8
Birth weight	950 g
Treatment	invasive mechanical ventilation for 2 days, non-invasive mechanical ventilation for 26 days, high-flow nasal cannula for 4 days, antibiotic therapy (ampicillin + gentamycin for 10 days)
MR exam	at term-equivalent age
Clinical outcome	discharged at a corrected age of 38 weeks and 6 days with a normal clinical status
**Patient 3**	
Clinical status	poor concentration, headache, forgetfulness, balance disorder, tinnitus, and double vision lasting seven months. occasional urinary incontinence for many years
Age	56 years
MR exam	56 years
Treatment	endoscopic ventriculostomy two months after MR imaging
Clinical outcome	all the symptoms regressed except tinnitus

MRI exams, at term-equivalent age and at age 2, were performed on a 3T MR device (Magnet Prisma^FIT^, Siemens, Germany) using a 64-channel head and neck coil. In addition to the standard T1 and T2 sequences, we also used a 3D T2 SPC sequence, which is sensitive to CSF movement artifacts (TR/TE = 3200/563 ms, resolution: 320 × 320, voxel size 0.8 × 0.8 × 0.8 mm), high-resolution 3D T2 space ZOOMit sequence (TR/TE = 1100/126 ms, resolution 164 × 320, voxel size: 0.5 × 0.5 × 0.5 mm), and high-resolution T1 MPRAGE sequence (TR/TE = 2300/3 ms, resolution = 256 × 256, voxel size = 1 × 1 × 1 mm).

**Figure 1:**
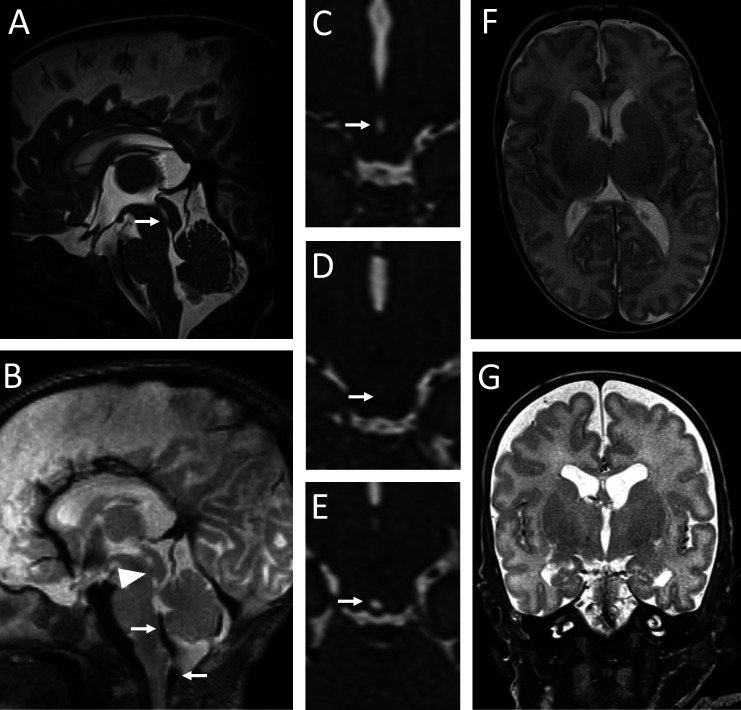
A magnetic resonance (MR) exam in Patient 1 at term-equivalent age. A mediosagittal T2 ZOOMit section shows a membrane in the caudal half of the mesencephalic aqueduct (arrow in **A**). Artifacts of cerebrospinal fluid movement are visible in the area of the foramen of Magendie and cranio-cervical junction (arrows in **B**), but are absent from the mesencephalic aqueduct (arrowhead in **B**). An axial T2 ZOOMit section through the mesencephalic aqueduct cranial to the obstruction site shows a maintained aqueduct lumen (arrow in **C**). An axial T2 ZOOMit section through the aqueductal membrane shows a complete obstruction of the mesencephalic aqueduct (arrow in **D**). An axial T2 ZOOMit section through the mesencephalic aqueduct caudal to the obstruction site shows a maintained aqueduct lumen (arrow in **E**). Axial (**F**) and coronal (**G**) T2 sections through the brain parenchyma show normal volume of the lateral ventricles and third cerebral ventricle.

MR scan at term-equivalent age showed a complete obstruction of the mesencephalic aqueduct by a membrane in the caudal half of the aqueduct (Figure 1A, Figure 1D). T2 SPC sagittal scans showed no artifacts indicative of CSF movement through the aqueduct (Figure 1B). In contrast, these artifacts were visible in the foramen of Magendie and cranio-cervical junction (Figure 1B). Axial and coronal scan through the brain parenchyma showed the third cerebral ventricle and lateral ventricles of appropriate size (Figures 1F and 1G), without radiological signs of triventricular hypertensive hydrocephalus (Evans’ index = 0.28).

**Figure 2:**
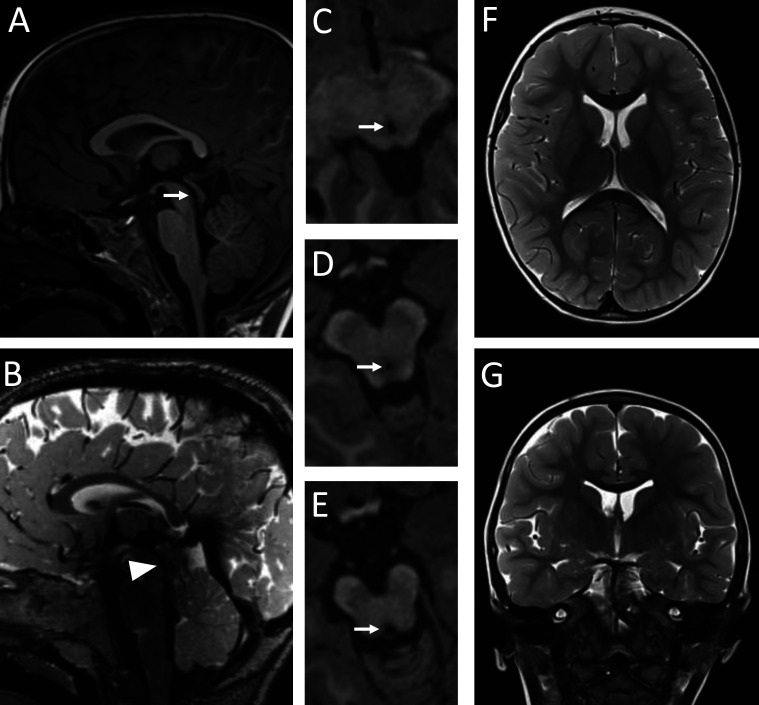
A magnetic resonance (MR) exam in Patient 1 at the age of 2 years. A mediosagittal MPRAGE section shows a passable mesencephalic aqueduct without an aqueductal membrane (arrow in **A**). Cerebrospinal fluid movement artifacts are visible on the T2 SPC sequence in the mesencephalic aqueduct (arrowhead in **B**). An axial MPRAGE section through the cranial part of the mesencephalic aqueduct shows a maintained aqueduct lumen (arrow in **C**). An axial MPRAGE section shows the patency of the mesencephalic aqueduct at the level where a membrane was previously visible (arrow in **D**). An axial MPRAGE section through the caudal part of the mesencephalic aqueduct shows a maintained aqueduct lumen (arrow in **E**). Axial (**F**) and coronal (**G**) T2 sections through the brain parenchyma show normal volume of the lateral ventricles and third cerebral ventricle.

At age 2, the scan (Figure 2) showed a passable lumen of the mesencephalic aqueduct (Figure 2A and 2D), with pronounced CSF movement artifacts (Figure 2B). The presented images indicate that a spontaneous rupture of the aqueduct membrane between the two MR exams established the CSF communication between the third and fourth ventricle.

## PATIENT 2

**Figure 3:**
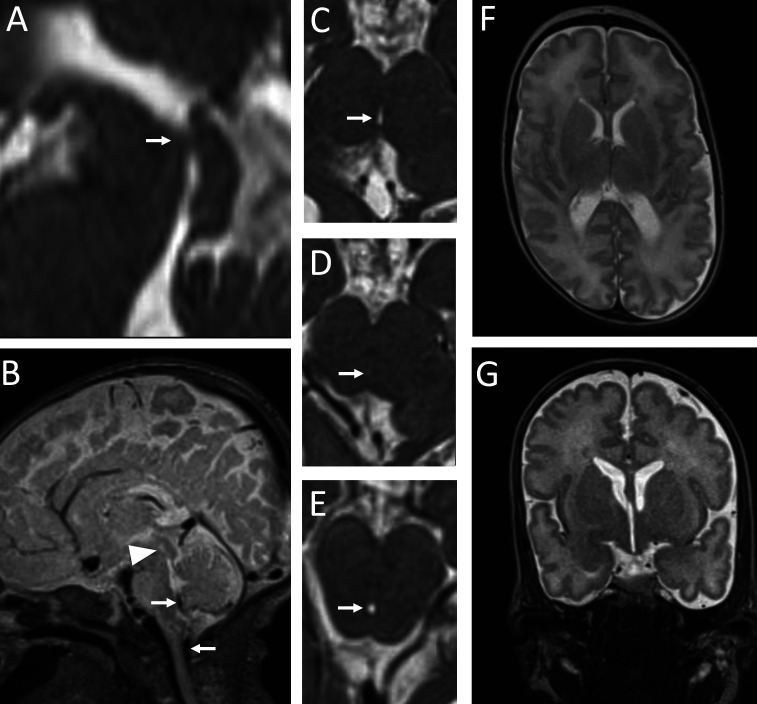
A magnetic resonance (MR) exam in Patient 2 at term-equivalent age. A mediosagittal T2 ZOOMit section shows an obstruction in the cranial portion of the mesencephalic aqueduct (arrow in **A**). Artifacts of cerebrospinal fluid movement are visible in the area of the foramen of Magendie and the cranio-cervical junction (arrows in **B**), but are completely absent from the mesencephalic aqueduct (arrowhead in **B**). An axial T2 ZOOMit section through the mesencephalic aqueduct cranial to the obstruction site shows a maintained aqueduct lumen (arrow in **C**). An axial T2 ZOOMit section at the obstruction level shows a completely obstructed aqueduct lumen (arrow in **D**). An axial T2 ZOOMit section through the mesencephalic aqueduct caudal to the obstruction site shows a maintained aqueduct lumen (arrow in **E**). Axial (**F**) and coronal (**G**) T2 sections through the brain parenchyma show adequate volume of the lateral ventricles and third cerebral ventricle.

Patient 2 was a preterm female infant born at a gestational age of 28 weeks and 6 days (birth weight 950 g, Apgar 6/8, head circumference 26 cm). After delivery, the patient developed respiratory distress syndrome requiring non-invasive and invasive mechanical ventilation. She also developed sepsis, but recovered completely after antibiotic therapy. The child was discharged at a corrected age of 38 weeks and 6 days, with a bodyweight of 2490 g (3 centiles), head circumference of 32 cm (5 centiles), and normal clinical status. MR exam was performed at term-equivalent age with the same MR sequences as described for Patient 1. The scan showed an obstruction of the cranial portion of the mesencephalic aqueduct (Figures 3A and 3D), which completely blocked the CSF communication between the third and fourth cerebral ventricle. T2 SPC sagittal scans showed no artifacts of CSF movement throughout the mesencephalic aqueduct. The artifacts were visible in the foramen of Magendie and craniocervical junction (Figure 3B). Axial and coronal sections through the brain parenchyma (Figure 3F and 3G) showed the third cerebral ventricle and lateral ventricles of appropriate size (Evans’ index = 0.26), without radiological and clinical signs of triventricular hypertensive hydrocephalus.

## PATIENT 3

Patient 3 was a 56-year-old man referred for MR exam due to poor concentration, headache, forgetfulness, balance disorder, tinnitus, and double vision lasting seven months. He also complained of occasional urinary incontinence for many years. The scanning was performed with the same 3T MR device as described for the two other patients. In addition to standard T1 and T2 cross-sections, high-resolution T2 CISS cross-sections (TR/TE = 5.3/2.4 ms, resolution 266 × 256; voxel 0.6 × 0.6 × 0.6 mm) were obtained through the area of the mesencephalic aqueduct.

**Figure 4:**
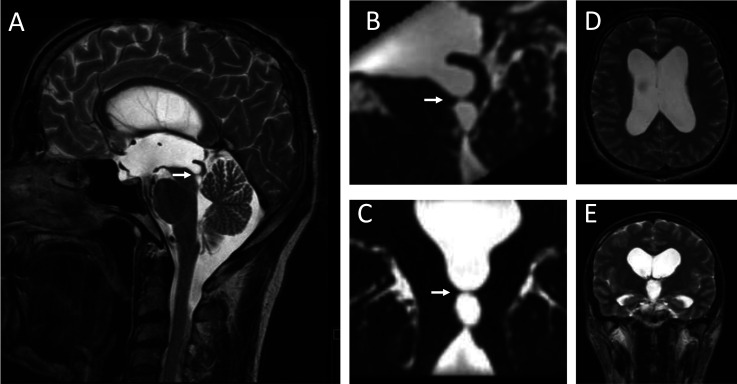
A magnetic resonance (MR) exam in Patient 3 at the age of 56 years. A mediosagittal T2 sequence shows an aqueductal membrane obstructing the mesencephalic aqueduct (arrow in **A**). 3D T2 CISS sections showing the aqueductal membrane completely dividing the central part of the mesencephalic aqueduct in the sagittal (**B**) and coronal planes (**C**). Axial (**D**) and coronal (**E**) T2 sections through the brain parenchyma show an enlarged third ventricle and lateral ventricles without transependymal cerebrospinal fluid edema that would indicate increased intraventricular pressure.

MR exam showed a complete obstruction of the central part of the mesencephalic aqueduct by an aqueductal membrane (Figure 4A, 4B, and 4C). Axial (Figure 4D) and coronal (Figure 4E) sections through the brain parenchyma showed an enlarged third cerebral ventricle and lateral ventricles (Evans’ index = 0.44) but no signs of transependymal CSF edema suggestive of intraventricular pressure increase. These clinical and neuroradiological findings are characteristic of late-onset aqueductal membranous occlusion (LAMO) hydrocephalus. Based on the clinical picture and neuroradiological findings, the patient underwent endoscopic ventriculostomy two months after MR imaging, which led to a regression of all symptoms, except tinnitus.

## DISCUSSION

In this study, we demonstrated in three patients how a blocked mesencephalic aqueduct could result in different neuroradiological and clinical outcomes, ranging from an entirely normal neuroradiological and clinical status to clinically and neuroradiologically verified hydrocephalus.

MRI scans performed in Patient 1 and Patient 2 at a neonatal age showed that it was possible for patients to have a completely obstructed mesencephalic aqueduct without showing clinical and radiological signs of triventricular hypertensive hydrocephalus. This finding is in contrast to the classical concept of CSF physiology, which postulates that about 500 mL of CSF a day is actively secreted (which is independent of intraventricular pressure) predominantly in the choroid plexuses of the lateral ventricles (8,9). This amount of CSF then passes through the mesencephalic aqueduct before being resorbed predominantly in the dural venous sinuses. Therefore, an obstruction of the mesencephalic aqueduct lasting only a few hours would necessarily change both the CSF volume and pressure within the third cerebral ventricle and lateral cerebral ventricles due to the accumulation of newly secreted CSF.

However, both patients' findings accord with the Bulat-Orešković-Klarica hypothesis, which assumes that the intracranial fluids exchange is controlled by osmotic and hydrostatic forces in the capillaries and interstitium and that it takes place on the capillary membrane along the entire nervous system (15-17). This means that depending on these forces CSF can be formed and resorbed at the same site. According to this hypothesis, there is no dominant CSF secretion site or a unidirectional movement. Numerous studies have shown that ventriculo-cisternal perfusion, as the only generally accepted method for determining CSF secretion, is a technical artifact. Namely, ventriculo-cisternal perfusion has been shown to measure CSF secretion even in experimental animals sacrificed with anesthetic overdose (18-21).

Animal experiments showed no expected increase in CSF pressure or ventricular dilation proximal to the obstruction site in the case of an aqueduct blockade lasting several hours (20). In addition, clinical observations demonstrated that long-term stenosis/obstruction of the aqueduct without hydrocephalus development was possible even when the patient was monitored for five years (21). Advanced neuroradiological methods (time-spatial inversion pulse) that visualize CSF movements showed only pulsatile, oscillatory movements of the CSF volume in all directions within the CSF system, with no net circulation in one direction (22).

Although Patients 1 and 2 had aqueduct obstruction without signs of hydrocephalus, a significant number of patients with aqueduct obstruction/stenosis do develop LAMO hydrocephalus, and some undergo endoscopic ventriculostomy (Patient 3) or placing of a drainage shunt due to a worsening clinical status (23-25). Despite obstruction/stenosis of the mesencephalic aqueduct in patients with LAMO hydrocephalus, hydrocephalus development cannot be explained by the classical concept. Namely, the classical concept cannot clarify why patients with aqueduct stenosis/obstruction often need surgery only later in life, as was the case in our Patient 3, although stenosis/obstruction had most likely been present for a long time, even before birth in some cases (26-28). Furthermore, these patients, despite the increase in CSF volume in the lateral ventricles and third ventricle, did not have an increased intraventricular pressure (Patient 3), as would be expected according to the classical concept.

Thus, the cases described in the current article do not accord with the generally accepted classical hypothesis, warranting a new explanation of the pathophysiological consequences of the mesencephalic aqueduct obstruction. Obstruction/stenosis of the mesencephalic aqueduct has been shown to decrease/interrupt the bidirectional oscillatory movement of CSF through the mesencephalic aqueduct that physiologically occurs during systole and diastole. This interruption changes the biomechanical load of the periventricular tissue (29). Prolonged exposure to altered biomechanical loading in different patients could lead to varying degrees of ventricular dilatation, ranging from normal findings to arrested hydrocephalus (30-32), or to neuroradiological and clinical presentation of hydrocephalus.

In patients with Chiari type 1 malformation, hydrocephalus often occurs due to the reduction/interruption of bidirectional CSF movement at the cranio-cervical junction. However, in Chiari type 1 malformation, assumed intracranial secretion, circulation, and CSF resorption are not disturbed (33,34). A similar pathophysiological mechanism is likely found in patients with spinal tumors who develop hydrocephalus (35).

In Patient 1, a spontaneous rupture of the aqueduct membrane established the CSF communication between the third and fourth brain ventricle. Clinical practice has shown that sometimes patients with an obstructed mesencephalic aqueduct experience spontaneous ventriculostomy, which establishes the CSF communication between the third ventricle and suprasellar cisterns (36-38). This observation could be explained by the previously mentioned changes in tissue biomechanical load due to a decreased/interrupted CSF bidirectional oscillatory movement.

Therefore, CSF obstruction at the aqueduct level causing ventricular dilatation without pressure rise is completely inexplicable by the classical hypothesis of CSF physiology. The described examples, however, accord with the Bulat-Orešković-Klarica hypothesis, which postulates that the obstruction of the mesencephalic aqueduct does not necessarily imply hydrocephalus development. This hypothesis defines hydrocephalus as a pathological state where CSF is excessively accumulated inside the cranial part of the CSF system, predominantly in one or more brain ventricles, as a consequence of impaired hydrodynamics of intracranial fluids between the CSF, brain (interstitial and intracellular fluids), and blood compartments (13).
